# Oral Nitrate Supplementation Differentially Modulates Cerebral Artery Blood Velocity and Prefrontal Tissue Oxygenation During 15 km Time-Trial Cycling in Normoxia but Not in Hypoxia

**DOI:** 10.3389/fphys.2018.00869

**Published:** 2018-07-16

**Authors:** Jui-Lin Fan, Nicolas Bourdillon, Philippe Meyer, Bengt Kayser

**Affiliations:** ^1^Wellington Medical Technology Group, Department of Surgery and Anaesthesia, University of Otago, Wellington, New Zealand; ^2^Centre for Translational Physiology, University of Otago, Wellington, New Zealand; ^3^Institute of Sports Sciences, University of Lausanne, Lausanne, Switzerland; ^4^Cardiology Service, Geneva University Hospital, Geneva, Switzerland

**Keywords:** cerebral tissue oxygenation, nitrate, NO bioavailability, exercise, hypoxia

## Abstract

**Background:** Nitrate is a precursor of nitric oxide (NO), an important regulator of cerebral perfusion in normoxic and hypoxic conditions. Nitrate supplementation could be used to improve cerebral perfusion and oxygenation during exercise in hypoxia. The effects of dietary nitrate supplementation on cerebral haemodynamics during exercise in severe hypoxia (arterial O_2_ saturation < 70%) have not been explored.

**Methods:** In twelve trained male cyclists, we measured blood pressure (BP), middle cerebral artery blood velocity (MCAv), cerebrovascular resistance (CVR) and prefrontal oxyhaemoglobin and deoxyhaemoglobin concentration (O_2_Hb and HHb, respectively) during 15 km cycling time trials (TT) in normoxia and severe hypoxia (11% inspired O_2_, peripheral O_2_ saturation ∼66%) following 3-day oral supplementation with placebo or sodium nitrate (0.1 mmol/kg/day) in a randomised, double-blinded manner. We tested the hypothesis that dietary nitrate supplementation increases MCAv and cerebral O_2_Hb during TT in severe hypoxia.

**Results:** During TT in normoxia, nitrate supplementation lowered MCAv by ∼2.3 cm/s and increased cerebral O_2_Hb by ∼6.8 μM and HHb by ∼2.1 μM compared to normoxia placebo (*p* ≤ 0.01 for all), while BP tended to be lowered (*p* = 0.06). During TT in severe hypoxia, nitrate supplementation elevated MCAv (by ∼2.5 cm/s) and BP (by ∼5 mmHg) compared to hypoxia placebo (*p* < 0.01 for both), while it had no effect on cerebral O_2_Hb (*p* = 0.98), HHb (*p* = 0.07) or PETCO_2_ (*p* = 0.12). Dietary nitrate had no effect of CVR during TT in normoxia or hypoxia (*p* = 0.19).

**Conclusion:** Our findings indicate that during normoxic TT, the modulatory effect of dietary nitrate on regional and global cerebral perfusion is heterogeneous. Meanwhile, the lack of major changes in cerebral perfusion with dietary nitrate during hypoxic TT alludes to an exhausted cerebrovascular reserve.

## Introduction

Nitric oxide (NO) is an important signalling molecule, responsible for regulating blood flow and tissue oxygen (O_2_) delivery under hypoxic conditions (Ignarro, 1989a,b; Kulandavelu et al., 2015). However, its role in the regulation of cerebral blood flow (CBF) during exercise is less clear. Due to its high metabolic demand and obligatory dependence on aerobic metabolism, the brain is particularly sensitive to any disruption in O_2_ supply, and adequate CBF is crucial for maintaining normal neurological function (Panickar and Anderson, 2011). Intense exercise in severe hypoxia (arterial O_2_ saturation < 70%) compromises brain O_2_ homeostasis by dramatically lowering cerebral tissue oxygenation (Subudhi et al., 2007, 2011). In partially acclimatized participants, data from Smith et al. (2014) indicates that cerebral O_2_ delivery is maintained during exercise at high altitude via increases in global CBF and changes in cerebral metabolic rate of O_2_. In altitude naïve participants, we found cerebral O_2_ delivery and prefrontal cortex tissue oxygenation to be reduced during exercise in severe hypoxia despite elevated middle cerebral artery blood velocity (MCAv) (Fan et al., 2013). Increasing NO bioavailability with dietary nitrate or NO-donor infusion modulates regional CBF response to sensory stimulation (Piknova et al., 2011; Aamand et al., 2013), which could account for the increase in CBF during exercise in hypoxia. Understanding the role of NO in the regulation CBF during exercise in normoxic and severe hypoxic conditions could provide novel insights into the compensatory mechanism(s) by which the cerebral O_2_ supply is maintained.

Studies have shown dietary nitrate supplementation to modulate cerebral haemodynamics during moderate exercise in normoxia (Thompson et al., 2014), while cerebral tissue oxygenation is unaffected during exercise in hypoxia with nitrate (Masschelein et al., 2012). But since these studies did not assess cerebral perfusion, the effect of increasing NO bioavailability on cerebral haemodynamics during exercise in severe hypoxia remain unclear. Previously, we assessed the effects of oral nitrate supplementation on pulmonary haemodynamics, muscle tissue oxygenation and performance during 15 km time trial cycling (TT) in normoxia and severe hypoxia (Bourdillon et al., 2015). The time-trial exercise paradigm was chosen as it provides more accurate simulation of physiological responses during competition and race performance (Foster et al., 1993; Palmer et al., 1996). We found dietary nitrate supplementation elicited greater muscle tissue desaturation during TT in hypoxia, while pulmonary haemodynamics and performance were unchanged. In this article, we present unpublished MCAv (as an estimate of global CBF) and prefrontal cortex tissue oxygenation data from that study. The aim of this article is to examine the role of NO bioavailability on cerebral haemodynamics during exercise in normoxia and severe hypoxia. Since the nitrate-nitrite-NO pathway has been reported to be enhanced in hypoxic conditions (Cosby et al., 2003; Shiva et al., 2007; Lundberg et al., 2008), we tested the following hypotheses: (1) oral dietary nitrate supplementation augments the MCAv response during TT in severe hypoxia; and (2) this increase in MCAv results in improved cerebral tissue oxygenation during exercise in severe hypoxia.

## Materials and Methods

### Participants

A power calculation was employed using a difference worth detection of a 4% improvement in 15-km TT cycling performance based upon previous studies (Jeukendrup et al., 2008; Fan et al., 2013). A sample size of twelve control volunteers was sufficient to give a power of 0.80 and an α of 0.05. Twelve male cyclists completed this study (age: 31 ± 7 years; weight: 73.5 ± 5.9 kg; maximal power output: 381 ± 36 W). All participants were low altitude residents, living in Geneva, Switzerland (389–450 m), non-smokers, with no previous history of cardiovascular, cerebrovascular, or respiratory disease and not taking any medication. All participants gave written informed consent prior to participation. The study was approved by the Research Ethical Committee of the University Hospitals of Geneva, Switzerland, and conformed to the standards set by the *Declaration of Helsinki*.

### Study Design

The study design has been previously described in detail (Bourdillon et al., 2015). Following a familiarization visit, which included a hypoxia sensitivity test and a maximal cardiopulmonary exercise test, the participants underwent two experimental treatments (placebo and nitrate), each consisting of three exercise sessions (six sessions total): (1) exercise echocardiography to assess pulmonary haemodynamics in normoxia and hypoxia: (2) 15 km TT in normoxia; and (3) 15 km TT in hypoxia. Data presented herein focuses on four of the six experimental testing sessions. Specifically: (1) 15 km TT in normoxia with placebo; (2) 15 km TT in normobaric hypoxia with placebo; (3) 15 km TT in normoxia with sodium nitrate; (4) 15 km TT in normobaric hypoxia with sodium nitrate. The 15 km TT sessions were separated by a ∼5-day rest period. The placebo/nitrate supplementations were carried out in a double-blind, randomized balanced crossover design, with a 5-day minimum washout period, while the normoxic/hypoxic conditions were administrated in a single-blinded, randomized balanced crossover manner. The TT sessions were conducted in an exercise physiology laboratory under similar laboratory conditions (temperature 24.1 ± 1.7°C, humidity 33 ± 5%, barometric pressure 721 ± 6 mmHg). Before each test, the participants refrained from caffeine for 12 h, and heavy exercise and alcohol for 24 h.

#### Oral Nitrate Supplementation

Three days prior to their first experimental session for each treatment conditions, the participants underwent oral supplementation with either sodium nitrate (nitrate: 0.1 mmol/kg/day) or placebo (sodium chloride: 0.1 mmol/kg/day) in identical capsules. This dosage has been shown to elevate plasma nitrate (${\mathrm{NO}}_{3}^{–}$) by ∼450% and plasma nitrite by ∼80% (Bescós et al., 2012). The participants continued the supplementation until they had completed all of the experimental sessions (i.e., a normoxic TT and a hypoxic TT). The average duration of the supplementation period was ∼8 days. The participants were instructed to avoid using mouthwash during the study period as it has been shown to reduce plasma nitrite/nitrate levels during dietary nitrate supplementation (Govoni et al., 2008). The participants were also instructed to avoid nitrate-rich foods during the entire study period in order to minimise the between-subject variations in dietary nitrate intake.

#### Experimental Setup

Throughout each experimental session, the participants wore a nose-clip and breathed through a mouthpiece attached to a low resistance one-way non-rebreathing valve (Hans-Rudolph 2700, Kansas City, KS, United States). The inspiratory side of the valve was connected to a gas mixing system (Altitrainer, SMTec, Nyon, Switzerland), from which they inspired either the normoxic or the hypoxic gas mixture. The fraction of inspired O_2_ (FIO_2_) was kept constant at either 0.21 (normoxia, ambient air) or 0.11 (hypoxia, inspired PO_2_ of ∼74 mmHg, equivalent of an altitude of ∼5,000 m). The participants breathed through the same circuit in all experimental sessions.

The TT sessions comprised 20 min of instrumentation with the participants breathing room-air while seated on a bicycle fitted to a Computrainer Pro Model trainer (RacerMate, Seattle, WA, United States). The trainer was calibrated according to the manufacturer’s instructions prior to each experimental session. The participants performed a 5-min self-selected warm-up exercise (heart rate < 120 bpm). After return to rest they were then switched to breathe from the gas mixing system for a 4-min post warm-up resting baseline collection. Immediately, following the baseline, the participants performed a 15 km TT as fast as possible. They were free to shift gears and choose pedaling rate. Constant feedback regarding the distance covered, but neither speed nor time, was provided on a computer screen together with a dynamic virtual reality environment showing a cycling avatar (Computrainer 3D software version 3.0, RacerMate). Two large ventilators were placed ∼60 cm in front of the participants and wind velocity was adjusted according to cycling speed.

### Measurements

#### Expired Nitric Oxide

Expired endogenous NO concentration was measured in duplicate during normoxic rest, at an expired rate of 50 ml/s (FeNO+, Medisoft, Sorinnes, Belgium).

#### Respiratory Variables

Breath-by-breath gas exchange and ventilatory flow were measured with a metabolic system (Medgraphics CPX, Loma Linda, CA, United States). Ventilation ($\dot{V}$E) was derived from the flow signal and expressed in l/min BTPS. Partial pressures of end-tidal O_2_ (PETO_2_) and CO_2_ (PETCO_2_) were derived from expired O_2_ and CO_2_ signals. The system was calibrated using a 3-L syringe and precision gas mixtures of known O_2_ and CO_2_ concentrations prior to each experimental session.

#### Cerebrovascular and Cardiovascular Variables

Beat-to-beat arterial blood pressure (BP) was monitored using finger plethysmography (Finometer MIDI, Finapres Medical Systems, Amsterdam, Netherlands). Peripheral O_2_ saturation (SPO_2_) was measured using earlobe pulse oximetry (Raical-7, Masimo Corporation, Irvine, CA, United States). Bilateral MCAv were measured using a 2-MHz pulsed Doppler ultrasound system (ST3, Spencer Technology, Seattle, WA, United States). The ultrasound probes were positioned over the temporal windows and held firmly in place with an adjustable headband. The signals were acquired at depths ranging from 43 to 54 mm. Signal quality was optimized and an M-mode screenshot was recorded to facilitate subsequent probe placements. Mean MCAv was calculated using the equation:

(1) $$\begin{array}{c} mean MCAv=(left MCAv+right MCAv)/2 \end{array}$$

Cerebrovascular resistance (CVR) was subsequently calculated using the equation:

(2) $$\begin{array}{c} \mathrm{CVR}=mean BP/mean MCAv \end{array}$$

Cerebral tissue oxygenation in the left prefrontal lobe was assessed by monitoring change in oxyhaemoglobin and deoxyhaemoglobin concentrations (O_2_Hb and HHb, respectively) and obtained with spatially resolved, continuous wave near infrared spectroscopy (NIRS, Artinis Oxymon, MKIII, Zetten, Netherlands). Source-detector spacing was set at 41 mm and data obtained from the optodes were used to monitor O_2_Hb and HHb, with a differential path-length factor (DPF) for the brain calculated using the formula (Duncan et al., 1995):

(3) $$\begin{array}{c} \mathrm{DPF}=4.99+0.067\times {\mathrm{age}}^{0.814} \end{array}$$

Cerebral total Hb (THb) was calculated using the following equation:

(4) $$\begin{array}{c} \mathrm{THb}=O_{2}\mathrm{Hb}\;+\;\mathrm{HHb} \end{array}$$

The NIRS data were expressed as absolute change.

#### Rate of Perceived Exertion (RPE)

During TT, the participants were asked to score their RPE on the 0–10 Borg scale every 3 km (Borg, 1982). The scale with descriptors was mounted in front of the participant at eye level. They were instructed to activate a handle bar mounted switch, activating the appropriate led indicator next to the descriptor corresponding to their RPE.

### Data Analysis

Resting values were obtained by averaging the data of the last 2 of the 4-min resting periods sitting on the stationary cycle just prior to the TTs. During the TTs, mean values for each variable were obtained from the entire 15 km. The effect of treatment on resting expired NO was assessed using paired student *t*-test (SPSS Statistics version 23, IBM Corporation, Armonk, NY, United States). The main effects of hypoxia and treatment on MCAv and cerebral oxygenation at rest and across the 15 km TT were assessed with mixed linear model analysis (SPSS Statistics version 23). For significant effects and interactions of hypoxia and treatment, *post hoc* tests were performed using Sidak’s adjustment for multiple comparisons (α-level of 0.05). In addition to *p*-values, Cohen’s *d*-values (effect size) are reported for hypoxia and treatment effects. Cohen’s *d*-value was calculated using the equation (Cohen, 1988):

(5) $$\begin{array}{c} d=(M_{condition1}\;-\;M_{condition2})/\sigma_{\mathrm{pooled}}^{2} \end{array}$$

where *M*_condition1_ and *M*_condition2_ are means of groups 1 and 2; $\sigma_{\mathrm{pooled}}^{2}$ is the standard deviation of the pooled data. The effect size is categorized as negligible (*d* < 0.2), small (*d* ≥ 0.2 and <0.5), medium (*d* ≥ 0.5 and <0.8), large (*d* ≥ 0.8 and <1.3), and very large (*d* ≥ 1.3). Data are reported as means ± SD in text, tables, and figures. Mean differences between condition and treatment were calculated from the average across the 15 km TT.

## Results

### Resting Variables (**Table 1**)

**Table 1:** Effect on hypoxia and dietary nitrate supplementation on resting cardiorespiratory and cerebral haemodynamics.

	Normoxia		Hypoxia		Main effect of (*p*-value)		
	Placebo	Nitrate	Placebo	Nitrate	Condition	Treatment	Interaction
***Cardiorespiratory***					
$\dot{V}$E (L/min)	16.4 ± 4.8	16.7 ± 2.6	17.5 ± 3.8	18.2 ± 3.2	0.20	0.72	0.59
PETO_2_ (mmHg)	102.5 ± 6.1	103.8 ± 4.4	43.9 ± 5.4	43.5 ± 4.0	**0.00^∗^**	0.44	0.38
PETCO_2_ (mmHg)	36.8 ± 4.6	37.4 ± 3.1	34.4 ± 3.8	34.1 ± 2.4	**0.00^∗^**	0.76	0.29
Mean BP (mmHg)	94.5 ± 13.9	92.6 ± 9.9	88.9 ± 11.3	95.2 ± 10.5	0.47	0.36	0.07
HR (b/min)	72.5 ± 11.9	68.1 ± 15.0	81.4 ± 13.8	82.3 ± 19.6	**0.00^∗^**	0.44	0.57
SPO_2_ (%)	97.3 ± 1.3	97.2 ± 0.9	83.4 ± 4.5	83.5 ± 2.9	**0.00^∗^**	0.97	0.86
**Cerebrovascular**					
MCAv (cm/s)	56.2 ± 8.2	53.9 ± 7.9	56.8 ± 11.6	57.9 ± 7.7	**0.00^∗^**	0.78	0.89
CVR (mmHg/cm/s)	1.78 ± 0.50	1.77 ± 0.38	1.66 ± 0.39	1.70 ± 0.33	0.20	0.84	0.69
O_2_Hb (μM)	0.6 ± 5.7	7.2 ± 8.4	–1.2 ± 2.5	0.8 ± 9.2	**0.02^∗^**	**0.01^∗^**	0.16
HHb (μM)	–2.8 ± 2.5	–0.9 ± 7.0	2.9 ± 1.9	4.4 ± 6.2	**0.00^∗^**	0.14	0.87
THb (μM)	–2.2 ± 8.1	6.2 ± 15.0	1.8 ± 3.1	5.2 ± 15.0	0.57	**0.03^∗^**	0.35

*$\dot{V}$*E, ventilation; PETO_*2*_; partial pressure of end-tidal O_*2*_; PETCO_*2*_, partial pressure of end-tidal CO_*2*_; BP, blood pressure; HR, heart rate; SPO_*2*_, peripheral O_*2*_ saturation; MCAv, middle cerebral artery blood velocity; CVR, cerebrovascular resistance; O_*2*_Hb, oxyhaemoglobin; HHb, deoxyhaemoglobin; THb, total haemoglobin. ^∗^p < 0.05. Data expressed as mean ± SD*.*

#### Expired NO

As previously reported (Bourdillon et al., 2015), paired *t*-test showed that dietary nitrate supplementation increased expired NO by ∼15.2 ppm (∼22%, *d* = 0.7, *p* = 0.01).

#### Cardiorespiratory

Resting $\dot{V}$E was unaffected by dietary nitrate (*d* = 0.1, *p* = 0.69) Hypoxia greatly lowered resting PETO_2_ by ∼59.5 mmHg (*d* = 2.0), SPO_2_ by ∼13.8% (*d* = 1.9) and PETCO_2_ by ∼2.9 mmHg (*d* = 0.7, *p* < 0.01 for all), and elevated resting HR by ∼11.5 b/min (*d* = 0.7, *p* < 0.01). Meanwhile, hypoxia had no effect on resting $\dot{V}$E (*d* = 0.4, *p* = 0.14), or BP (*p* = 0.47).

Dietary nitrate tended to have a greater effect on mean BP in hypoxia compared to normoxia (main effect: *p* = 0.36, interaction: *p* = 0.07), while no treatment effect was observed in any other cardiorespiratory variables (**Table 1**). *Post hoc* analysis showed a trend for higher resting BP (by ∼6.5 mmHg, *d* = 0.6, *p* = 0.06) with dietary nitrate in hypoxia compared to hypoxic placebo value, while no such differences were observed in normoxia (*d* = 0.2, *p* = 0.51). While hypoxia *per se* had no effect on resting mean BP (*p* = 0.47). We found no changes in resting HR with dietary nitrate (*p* = 0.44), while hypoxia elevated it by ∼11.5 b/min irrespective of treatment (*d* = 0.7, *p* < 0.01).

#### Cerebrovascular

Acute hypoxia increased MCAv by ∼6% (*d* = 0.4, *p* < 0.01) and lowered resting O_2_Hb by ∼4.1 μM (*d* = 0.6, *p* = 0.02) and elevated resting HHb by ∼5.6 μM when compared to normoxia (*d* = 1.0, *p* < 0.01) but had no effect on CVR (*d* = 0.2, *p* = 0.20). Resting THb was unaltered with hypoxia (*p* = 0.57). Dietary nitrate increased resting O_2_Hb by ∼4.2 μM (*d* = 0.6, *p* = 0.01) and THb by ∼6.0 μM (*d* = 0.5, *p* = 0.03) compared to placebo treatment, but had no effect of resting HHb (*d* = 0.3, *p* = 0.14). Dietary nitrate had no significant effects on resting MCAv or CVR (*p* = 0.78 and 0.84, respectively).

### Exercise

As previously reported (Bourdillon et al., 2015), hypoxia decreased TT performance (i.e., time-to-completion) by ∼22% (*d* = 1.6, *p* < 0.01), while there was no significant effect of dietary nitrate on TT performance (*d* = 0.2, *p* = 0.36).

#### Cardiorespiratory Variables

**FIGURE 1:**
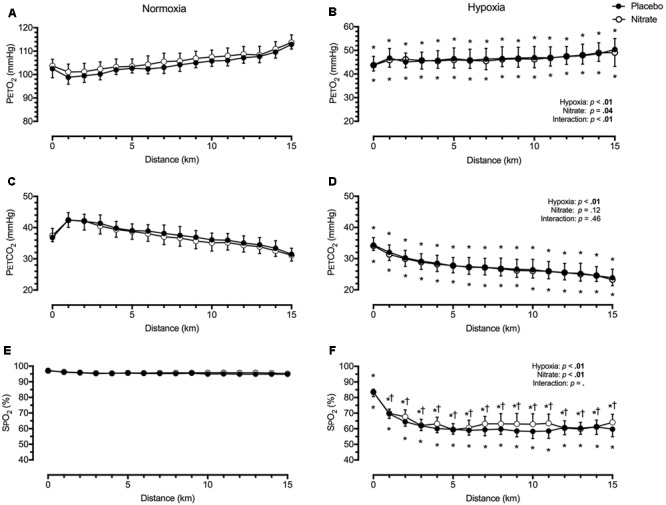
Effect of dietary nitrate supplementation on end-tidal gases and peripheral O_2_ saturation during 15 km TT in normoxia and hypoxia. PETO_2_, partial pressure of end-tidal O_2_ (**A** and **B**); PETCO_2_, partial pressure of end-tidal CO_2_ (**C** and **D**); SPO_2_, peripheral O_2_ saturation (**E** and **F**). † different from placebo, *p* < 0.05; ^∗^ different from normoxia, *p* < 0.05. Data expressed as mean ± SD.

The respiratory and BP data have been previously reported (Bourdillon et al., 2015). Irrespective of treatment condition, hypoxia elevated $\dot{V}$E by ∼8.2 L/min (*d* = 0.2) during TT compared to normoxia, while PETCO_2_ (by ∼10.1 mmHg, *d* = 1.5), PETO_2_ (by ∼58.3 mmHg, *d* = 2.0) and SPO_2_ (by ∼33.6%, *d* = 1.9) were all greatly lowered (*p* < 0.01 for all, **Figure 1**). Dietary nitrate caused small elevations in $\dot{V}$E by ∼6.3 L/min during TT compared to both normoxic and hypoxic placebo conditions (*d* = 0.2, *p* < 0.01). As a result, PETO_2_ was elevated by ∼1.7 mmHg with nitrate during TT in normoxia (*d* = 0.3, *p* < 0.01), but not during TT in hypoxia (*p* = 0.37, **Figure 1**). Despite the increase in $\dot{V}$E with dietary nitrate, PETCO_2_ was unaffected during TT in normoxia and hypoxia compare placebo conditions (*d* < 0.1, **Figure 1**, *p* = 0.12). Dietary nitrate improved SPO_2_ during TT in hypoxia by ∼2.8% (*d* = 0.4, *p* < 0.01), but had no significant effect on SPO_2_ during TT in normoxia when compared to placebo values (*p* = 0.40, **Figure 1**).

**FIGURE 2:**
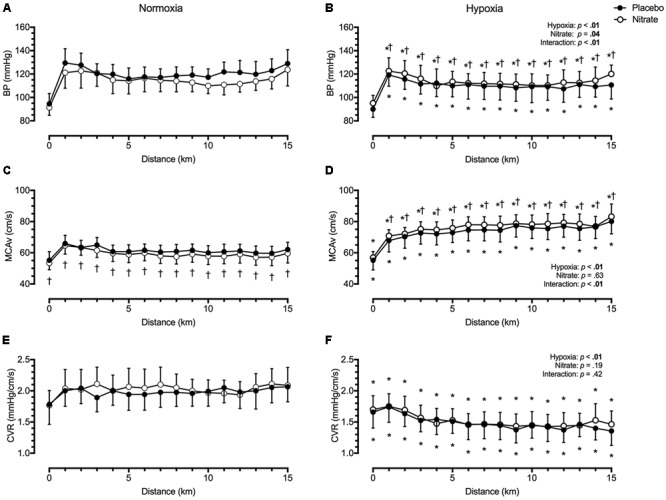
Effect of dietary nitrate supplementation on blood pressure, middle cerebral blood velocity and cerebrovascular resistance during 15 km TT in normoxia and hypoxia. BP, blood pressure (**A** and **B**); MCAv, middle cerebral blood velocity (**C** and **D**); CVR, cerebrovascular resistance (**E** and **F**). † different from placebo, *p* < 0.05; ^∗^ different from normoxia, *p* < 0.05. Data expressed as mean ± SD.

We found mean BP to be lower by ∼9.1 mmHg during TT in hypoxia compared to during TT in normoxia (*d* = 0.6, *p* < 0.01, **Figure 2**). However, this was attenuated with dietary nitrate (interaction: *p* < 0.01). *Post hoc* analysis showed that dietary nitrate elevated mean BP by ∼5.3 mmHg during TT in hypoxia (*d* = 0.3, *p* < 0.01). In contrast, dietary nitrate tended to lower mean BP by ∼2.0 mmHg during TT in normoxia (*d* = 0.2, *p* = 0.06).

#### Cerebrovascular Variables

Irrespective of treatment, hypoxia greatly elevated mean MCAv by ∼15.3 cm/s during TT compared to normoxia (*d* = 1.3, *p* < 0.01, **Figure 2**). This MCAv increase was mediated by a reduction in CVR with hypoxia by ∼0.51 mmHg/cm/s compared to normoxic values (*d* = 1.2, *p* < 0.01, **Figure 2**). Dietary nitrate elicited a differential effect on mean MCAv during normoxic and hypoxic exercise (interaction: *p* < 0.01). During TT in normoxia, mean MCAv was lower by ∼2.3 cm/s with dietary nitrate compared to placebo values (*d* = 0.3, *p* = 0.01). During TT in hypoxia, mean MCAv was higher by ∼2.5 cm/s with dietary nitrate compared to placebo values (*d* = 0.2, *p* < 0.01). Despite these changes, we found no significant effect of treatment on CVR (*d* < 0.1, *p* = 0.19).

**FIGURE 3:**
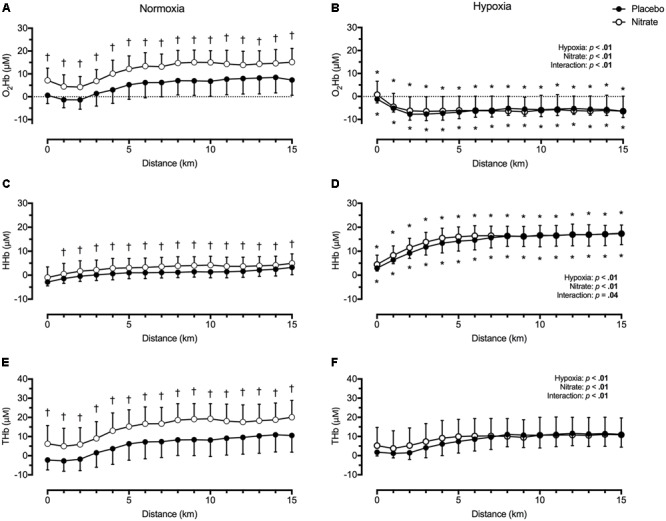
Effect of dietary nitrate supplementation on cerebral tissue oxygenation of the prefrontal cortex during 15 km TT in normoxia and hypoxia. O_2_Hb, oxyhaemoglobin concentration (**A** and **B**); HHb, deoxyhaemoglobin concentration (**C** and **D**); THb, total haemoglobin concentration (**E** and **F**). ^†^different from placebo, *p* < 0.05; ^∗^ different from normoxia, *p* < 0.05. Data expressed as mean ± SD.

Regardless of treatment, hypoxia greatly lowered O_2_Hb, by ∼14.8 μM (*d* = 1.3), greatly elevated HHb, by ∼12.8 μM (*d* = 1.5), and slightly elevated THb, by ∼2.0 μM (*d* = 0.2) during TT compared to normoxia (*p* < 0.01 for all, **Figure 3**). Dietary nitrate supplementation elicited a greater effect on prefrontal tissue oxygenation during normoxic exercise compared to hypoxic exercise (interaction: *p* < 0.05). During TT in normoxia, dietary nitrate elevated prefrontal O_2_Hb by ∼6.8 μM (*d* = 0.7), HHb by ∼2.1 μM (*d* = 0.4), THb by ∼9.0 μM (*d* = 0.6, *p* < 0.01 for all, **Figure 3**). During TT in hypoxia, dietary nitrate had no effect on prefrontal HHb (*d* = 0.1, *p* = 0.07), O_2_Hb (*p* = 0.98) and THb (*p* = 0.38) compared to placebo.

#### Rate of Perceived Exertion

The participants RPE during TT, as indicated by the Borg score, was elevated by ∼0.8 in hypoxia compared to normoxic TT (*d* = 0.4, *p* < 0.01), but was unaffected with dietary nitrate (*d* < 0.1, *p* = 0.69).

## Discussion

We studied the effects of dietary nitrate supplementation (∼7.3 mmol ${\mathrm{NO}}_{3}^{–}$/day) on MCAv and prefrontal tissue oxygenation during intense exercise in normoxia and hypoxia (SPO_2_ ∼66%). We found dietary nitrate supplementation marginally lowered MCAv and moderately elevated prefrontal tissue oxygenation during TT in normoxia. In agreement to our first hypothesis, dietary nitrate elevated MCAv during TT in hypoxia, albeit by a marginal extent. But in contrast to our second hypothesis, this did not translate into improved prefrontal tissue oxygenation. Since CVR during TT in normoxia and hypoxia were unchanged with dietary nitrate supplementation, our findings indicate that the changes in MCAv were mediated by the effect of dietary nitrate on BP during exercise.

### Nitric Oxide and Cerebral Haemodynamics in Normoxia

Nitric oxide is an important signalling molecule involved in the regulation of brain vascular tone (McPherson et al., 1995; Rifkind et al., 2007; Peebles et al., 2008; Terpolilli et al., 2012). Using functional magnetic resonance imaging (MRI), Presley et al. (2011) found 2-days of high nitrate diet (12.4 mmol/day) selectively improved prefrontal cortex perfusion without altering global CBF. In a group of trained male participants, we assessed the effects of dietary nitrate supplementation on MCAv and prefrontal NIRS signals during TT, as indices of global perfusion and tissue oxygenation, respectively. We found dietary nitrate lowered both MCAv by a small extent (*d* = 0.3) and tended to lower mean BP (*d* = 0.2) during TT in normoxia (**Figure 2**). Meanwhile, dietary nitrate elevated PETO_2_ during normoxic TT (*d* = 0.3) but had negligible effect on PETCO_2_ (*d* < 0.1). Since changes in arterial O_2_ content (as determined by SPO_2_ and PETO_2_) result in reciprocal changes in CBF to maintain constant cerebral O_2_ delivery (Jones et al., 1981), this increase in PETO_2_ (mediated by increased ventilation) could explain some of the decline in MCAv during normoxic TT with dietary nitrate (**Figure 1**).

Before discussing the effects of dietary nitrate on cerebral haemodynamics, one must first acknowledge the major determinants of CBF. Blood flow through the brain can be described by Ohm’s law, which states that:

$$\begin{array}{c} Q=\;\frac{\Delta P\;\;}{R\;} \end{array}$$

where Q is CBF, Δ*P* is the cerebral perfusion gradient, which is calculated from the difference between BP and downstream pressures of the cerebral circulation (i.e., intracranial pressure, central venous pressure), while *R* is the resistance of the entire cerebral circulation, which is under influence of neural, humoral, metabolic and physical factors (Faraci and Heistad, 1990; Edvinsson and Krause, 2002). Assuming unaltered downstream pressures, our finding indicates that dietary nitrate does not affect CVR during normoxic TT (**Figure 2**). In untrained females, Bond et al. (2013) found acute beetroot juice ingestion (∼6.2 mmol ${\mathrm{NO}}_{3}^{–}$) elevated MCAv and lowered systolic BP during steady-state exercise in normoxia (40 and 80% peak workload). They further found the CVR to be lowered with dietary nitrate (i.e., ↓ BP and ↑ MCAv). The discrepant MCAv findings between those by Bond et al. (2013) and the present study are difficult to reconcile, despite both studies demonstrating reduced system vascular resistance with dietary nitrate. Dietary nitrate supplementation has been shown to be less effective in males (Kapil et al., 2010), and those with high aerobic fitness (Wilkerson et al., 2012; Porcelli et al., 2014). Therefore, differences in sex, fitness and nitrate dosage (i.e., ∼6.2 mmol at 2 h prior to exercise vs. ∼7.3 mol/day) could account for some of the disparities between our study and those by Bond et al. (2013). We speculate that the combination of a blunted response in male participants and a lower dose of dietary nitrate could account for the lack of change in CVR in our study (see *methodological considerations*).

In agreement with findings by Presley et al. (2011), we found dietary nitrate moderately elevated resting prefrontal O_2_Hb and THb (*d* = 0.6–0.7, **Table 1**), which remained elevated during TT in normoxia (**Figure 3**). In addition, prefrontal HHb was selectively elevated during TT in normoxia with dietary nitrate compared to placebo. Together with previous findings, our data indicate the dietary nitrate supplementation selectively improved perfusion and tissue oxygenation to the prefrontal cortex at rest and during exercise in normoxia, without large changes in MCAv. One possible explanation for the concurrent decrease in MCAv and increase in prefrontal tissue oxygenation is an enhanced neurovascular coupling with dietary nitrate supplementation. Under physiological conditions, functional MRI studies have found regional perfusion typically outstrips metabolic requirement by two–sixfold during sustained neuronal activation (Fox and Raichle, 1986; Lin et al., 2010; Vafaee et al., 2012). Acute dietary nitrate ingestion (∼5.5 mmol, 90 min prior) enhances prefrontal-NIRS response during the initial phase of cognitive tasks (Wightman et al., 2015). Meanwhile, a 3-day dietary nitrate supplementation (0.1 mmol/kg/day) reduced both the lag and the *amplitude* of CBF response (assessed using functional MRI) in the primary visual cortex during visual stimulation (Aamand et al., 2013). These findings support an enhanced neurovascular coupling with dietary nitrate, mediated by higher cortical tissue oxygenation and lower CBF response during cortical activation. Using positron emission tomography, Hiura et al. (2014, 2018) found moderate steady-state cycling (∼50% estimated max HR) selectively increased regional blood flow to cerebellar vermis and primary sensorimotor cortex, insular cortex and brain stem. Since prefrontal cortical tissue oxygenation changes during exercise reflect those of the sensorimotor cortex (Subudhi et al., 2009), an enhanced neurovascular coupling with dietary nitrate could account for the elevated prefrontal tissue oxygenation and reduced MCAv during TT in normoxia.

### Nitric Oxide and Cerebral Haemodynamics in Hypoxia

Hypoxia presents a unique and significant challenge to cerebral O_2_ balance and is well-known to detrimentally impair aerobic capacity (Calbet and Lundby, 2009; Ainslie and Subudhi, 2014). During intense exercise, severe hypoxia dramatically lowers cerebral tissue oxygenation despite concurrent increases in cerebral perfusion (Subudhi et al., 2007; Peltonen et al., 2009; Fan and Kayser, 2013; Fan et al., 2013). Several studies have reported an inverse relationship between cerebral tissue desaturation and exercise performance, leading some to conclude that cerebral tissue desaturation could be a limiting factor on performance in severe hypoxia [(Kjaer et al., 1999; Amann et al., 2006, 2007; Rasmussen et al., 2010), see (Fan and Kayser, 2016) for review]. Nitric oxide is involved in regulating blood vessel tone and tissue oxygenation (Ignarro, 1989a,b; Kulandavelu et al., 2015). Since NO-induced vasodilation is facilitated by the presence of deoxyhaemoglobin (Brooks, 1937; Cosby et al., 2003), it has been postulated that upregulation of the nitrate-nitrite-NO pathway could be protective against hypoxic insults by selectively increasing blood flow to regions where O_2_ supply is limited (Lundberg et al., 2008).

Bailey et al. (2017) found exercise and hypoxia independently and additively increased net nitrite consumption and red blood cell NO metabolite formation (predominantly nitrosylhaemoglobin) in the brain and the working limb. They found linear relationships between the net cumulative bioactive NO metabolite formation and the increase in femoral and cerebral blood flow. These findings provide compelling evidence that deoxyhaemoglobin-mediated nitrite reduction and associated NO production is responsible for hypoxic vasodilation during exercise in hypoxia. We hypothesised that an enhanced NO bioavailability would elevate MCAv during TT in hypoxia, thereby improving cerebral tissue oxygenation and possibly performance. But there is limited data on the effect of dietary nitrate on cerebral tissue oxygenation in hypoxia. During TT exercise at ∼3,000 m and ∼4,300 m (SPO_2_ ∼80% and ∼70%, respectively), Shannon et al. (2017) found beetroot juice supplementation lowered cerebral O_2_Hb and THb, and elevated cerebral HHb compared to placebo treatment. In contrast, Masschelein et al. (2012) found no improvements in prefrontal cortex tissue oxygenation with dietary nitrate during steady-state and incremental cycling at simulated 5,000 m (SPO_2_ ∼64%). In our study, we found both MCAv and mean BP were elevated by similar magnitude (*d* = 0.2–0.3) during TT in severe hypoxia (SPO_2_ ∼62%) with dietary nitrate compared to placebo, resulting in an unchanged CVR (**Figure 2**). Given the vasodilatory effects of dietary nitrate during normoxic exercise (Bond et al., 2013; Lee et al., 2015), this increase in mean BP during hypoxic TT is likely mediated by an increased cardiac output with dietary nitrate. Previously, we found dietary nitrate supplementation improved right ventricular-atrial pressure gradient (index of right heart contractility) during steady-state hypoxic exercise (Bourdillon et al., 2015). Such improvement in cardiac function with dietary nitrate could account for the increases in mean BP and MCAv during exercise in severe hypoxia.

Despite a lack of change in end-tidal gases during hypoxic TT with dietary nitrate, we found SPO_2_ to be moderately elevated (*d* = 0.4) – mediated by a small increase in $\dot{V}$E (*d* = 0.2). Since no differences in prefrontal NIRS signals or time-to-completion were observed between dietary nitrate and placebo treatment during hypoxic TT (**Figure 3**), we conclude that increases in MCAv and SPO_2_ were too small to have any significantly impact on cerebral O_2_ delivery and performance. The lack of change in tissue oxygenation and performance was unexpected, considering prefrontal tissue oxygenation was improved with dietary nitrate during normoxic TT (**Figure 3**). This disparity could be accounted by a loss of cerebrovascular reserve with exercise in severe hypoxia.

In altitude naïve participants, we previously found TT exercise in severe hypoxia impaired cerebral vasodilatory response to hypercapnia — a potent cerebral vasodilatory stimuli (Fan et al., 2013). We speculated that the cerebrovascular reserve is exhausted during exercise in severe hypoxia (i.e., maximal cerebral vasodilation reached), thus any additional vasodilatory stimuli would not result in additional increases in cerebral perfusion. In addition, we observed a progressive rise in MCAv and Borg score during TT in hypoxia, in absence of significant changes in BP or exercise workload (Fan et al., 2013). Since exercise selectively increases regional CBF to the sensorimotor cortex (Hiura et al., 2014, 2018) – presumably due to greater afferent feedback, we speculated that the progressive increase in RPE (and associated greater sensorimotor cortex activation) could account for the progressive rise in MCAv during TT in hypoxia. In the present study, we found RPE to be elevated during TT in hypoxia but unaffected with dietary nitrate. Given an enhanced neurovascular coupling with nitrate (Aamand et al., 2013; Wightman et al., 2015), we would have expected to see changes in MCAv and prefrontal NIRS similar to that observed during normoxic TT. Instead, we observed a small increase in MCAv with dietary nitrate supplementation accompanied by unchanged prefrontal tissue oxygenation during hypoxic TT (**Figures 2, 3**).

### Adequate O_2_ Supply During Hypoxic Exercise?

The cerebrovascular response to hypoxia is heterogeneous within the cerebral circulation, with the posterior and inferior circulation more sensitive to hypoxia compared to anterior regions (Willie et al., 2012; Subudhi et al., 2014), presumably to preserve vital homeostatic control centres in those brain regions. There is emerging evidence that the energetically expensive brain has evolved ‘selfishly’ to place the utmost priority on maintaining its own blood supply (Paton et al., 2009). According to this ‘selfish brain’ hypothesis, in situations where cerebral O_2_ delivery is threatened such as severe hypoxia, CBF will rise to compensate (Smith et al., 2014; Subudhi et al., 2014). During TT in severe hypoxia (SPO_2_ ∼65%), we have previously found cerebral O_2_ delivery (derived from MCAv and capillary O_2_ content) is maintained to normoxic values, via increases in MCAv (Fan et al., 2013). Our finding was subsequently confirmed by Smith et al. (2014), who found the increase in cerebral perfusion (assessed using volumetric measures of CBF) during exercise at 5,050 m (SPO_2_ ∼75%) sufficiently compensates for the drop in arterial O_2_ content, thereby maintaining cerebral O_2_ delivery to normoxic values. Therefore, an already optimised cerebral perfusion and/or exhausted cerebrovascular reserve could potentially explain the lack of any large improvements in MCAv and prefrontal NIRS during hypoxic TT with dietary nitrate.

### Methodological Considerations

An important consideration when interpreting our data is the possible extracranial influence on the prefrontal NIRS signal. Using headband cuff and face cooling, reductions in skin blood flow have been shown to reduce prefrontal O_2_Hb, while prefrontal HHb was relatively unaffected (Miyazawa et al., 2013; Hirasawa et al., 2015, 2016). Meanwhile, the relative contribution of skin blood flow on brain NIRS signal was reduced when the source-detector distance was increased from 15 to 30 mm (Hirasawa et al., 2015, 2016). Data from Kohri et al. (2002) demonstrate that the relative contributions of cerebral tissue on cerebral NIRs signal at source-detector distances of 20, 30, and 40 mm are 33, 55, and 69%, respectively. These findings clearly highlight extracranial blood flow influence on NIRS-derived cerebral O_2_Hb, which can be reduced with increased source-detector distance. An increased cutaneous vascular conductance with dietary nitrate could account for the elevated cerebral O_2_Hb during normoxic TT. It should be acknowledged that, dietary nitrate supplementation does not appear to influence forearm cutaneous vascular conductance response to exercise in heat (Amano et al., 2018), nor alter cutaneous reactive hyperaemic response (Wong et al., 2017). Moreover, the use of a source-detector distance of 41 mm in our setup reduces the extracranial influence on prefrontal NIRS signals.

We have previously addressed the limitations of using MCAv to represent global CBF during both exercise and hypoxia (Fan and Kayser, 2013; Fan et al., 2013). More recently, Smith et al. (2014) found close agreement between magnitude of MCAv and global CBF changes (as assessed using Fick’s principle) to progressive steady-state exercise at sea-level and at 5,050 m. Nevertheless, the possible influences of oral nitrate on MCA diameter must also be considered when interpreting our MCAv data. Administration of glyceryl trinitrate (1 mg), a NO-donor, has been shown to decrease MCAv without affecting CBF, as assessed with single photon emission computed tomography (Dahl et al., 1989). The authors attributed the reduction in MCAv to dilatation of the MCA. At 0.3–6 mg/kg, administration *N*^G^-monomethyl-L-arginine infusion (L-NMMA, a NO synthase inhibitor) does not significantly alter resting MCAv or CBF (assessed using gradient-echo phase contrast MRI) (Van Mil et al., 2002; Hjorth Lassen et al., 2003). Between 1 and 10 mg/kg, L-NMMA reduces internal carotid artery blood flow in a dose-dependent manner without altering MCAv, suggesting constriction of MCA (White et al., 1998). These findings led Hjorth Lassen et al. (2003) to attribute the apparent MCA constriction with L-NMMA to a physiological response to reduced CBF rather than a direct influence of NO on MCA basal tone. In the present study, we found dietary nitrate had no effect on MCAv at rest (**Table 1**), while it was lowered during TT in normoxia (**Figure 2**). Therefore, it is possible that the observed reduction in MCAv was due to dilatation of MCA *per se* independent of changes in global CBF. Moreover, if dietary nitrate increased MCA diameter, then MCAv would likely *underestimate* the magnitude of the increase in cerebral perfusion estimates during TT in hypoxia.

In the present study, we assessed the effects of dietary nitrate on cerebral tissue oxygenation in a group of male cohorts during 15 km TT. Meta-analysis found nitrate supplementation moderately improved time-to-exhaustion performance and elicited small improvements on time-trial and incremental exercise tests (Hoon et al., 2013; McMahon et al., 2017). Moreover, the beneficial effects of dietary nitrate supplementation appeared to be limited to non-athletes (Campos et al., 2018). Since a majority of the studies investigated the performance benefits of dietary nitrate in male participants, sex differences to dietary nitrate supplementation have been largely overlooked. Kapil et al. (2010) found lower sensitivity to potassium nitrate supplementation in men compared to women, which they attributed to a lower baseline plasma nitrite in their male participants. Meanwhile, unpublished data from our laboratory indicates dietary nitrate supplementation enhanced cerebrovascular function in men but not women. Therefore, the efficacy of dietary nitrate supplementation appears to be related to an individual’s baseline nitrate concentration, which is influenced by sex and aerobic fitness.

## Conclusion

Dietary nitrate supplementation caused a small reduction in MCAv and moderately elevated prefrontal tissue oxygenation during time-trial exercise in normoxia. Since BP tended to be concurrently lowered by a similar magnitude to the MCAv change, our data alludes to a preserved CVR with nitrate supplementation. We attribute the cerebral haemodynamic changes to the heterogeneous effects of dietary nitrate on global and prefrontal perfusion. Meanwhile, dietary nitrate elicited small elevations in both BP and MCAv during time trial exercise in hypoxia, without altering prefrontal tissue oxygenation. We speculate that this may be due to an exhaustion of the cerebral dilatory capacity during exercise in severe hypoxia.

## Author Contributions

BK, J-LF, and PM contributed to the conception and design of the study. J-LF and NB performed the data collection. J-LF led the analysis and interpretation of the data, drafted the manuscript, and prepared the figures. BK contributed to the revision of the manuscript. All authors approved the final version of the manuscript.

## Conflict of Interest Statement

The authors declare that the research was conducted in the absence of any commercial or financial relationships that could be construed as a potential conflict of interest. The reviewer PB and handling Editor declared their shared affiliation.
